# Elucidation of novel turnagainolides and their biosynthetic gene cluster in *Bacillus subtilis*

**DOI:** 10.1128/aem.02574-24

**Published:** 2025-04-25

**Authors:** Peng Li, Shuang Han, Min Wang, Xuejiao Zhang, Shuai Zhi, Meiling Jin, Jouni Jokela, Shan He, Liwei Liu

**Affiliations:** 1 Li Dak Sum Yip Yio Chin Kenneth Li Marine Biopharmaceutical Research Center, Health Science Center, Ningbo University 74633, Ningbo, Zhejiang, China; 2 College of Food Science and Engineering, Ningbo University 47862, Ningbo, Zhejiang, China; 3 College of Biotechnology, Tianjin University of Science and Technology 534786, Tianjin, China; 4 College of New Materials and Chemical Engineering, Beijing Institute of Petrochemical Technology 34735, Beijing, China; 5 National-Regional Joint Engineering Research Center for Soil Pollution Control and Remediation in South China, Guangdong Key Laboratory of Integrated Agro-environmental Pollution Control and Management, Institute of Eco-environmental and Soil Sciences, Guangdong Academy of Sciences 514144, Shenyang, China; 6 School of Public Health, Ningbo University 631344, Ningbo, Zhejiang, China; 7 Chinese Academy of Sciences (CAS) Key Laboratory of Quantitative Engineering Biology, Chinese Academy of Sciences Shenzhen Institute of Advanced Technology 85411 , Shenzhen, Guangdong, China; 8 Department of Microbiology, University of Helsinki 3835, Helsinki, Finland; 9 Ningbo Institute of Marine Medicine, Peking University, Ningbo, Zhejiang, China; Kyoto University, Kyoto, Japan

**Keywords:** *Bacillus*, depsipeptide, biosynthesis, turnagainolide, bioactivity, secondary metabolite

## Abstract

Turnagainolides represent a unique class of cyclic depsipeptides characterized by the presence of a rare (*E*)-3-hydroxy-5-phenylpent-4-enoic acid (Hppa) residue, exhibiting diverse bioactivities. While previous studies have identified turnagainolides and their congeners from various microorganisms, including *Microascus*, *Bacillus*, *Arthrobacter*, and *Streptomyces*, their biosynthetic gene cluster and pathways remained elusive. Here, we uncovered four novel compounds, turnagainolide congeners D-G (**3–6**), and two known compounds, turnagainolides A-B (**1, 2**), from *Bacillus subtilis* LP. Their chemical structures were elucidated through a combination analysis of LC-MS analysis, NMR spectroscopy, and the Mosher derivatization technique. To investigate their biosynthetic gene cluster, comprehensive genome sequencing, phylogenetic analysis, and anti-SMASH-based prediction were conducted, and gene knockout experiments confirmed the correlation between the *tur*-BGC and the biosynthesis of these compounds. The alignment of protein sequences encoded by *tur*-BGC against public protein databases revealed homologous proteins exclusively in *Bacillus* species. These findings not only expand the chemical diversity of cyclic peptides in *Bacillus* but also provide critical insights into the biosynthetic pathway of turnagainolides and their evolutionary lineage.

**IMPORTANCE:**

Microbial natural products represent an invaluable resource in drug discovery, providing a vast reservoir of structurally and functionally diverse compounds with promising therapeutic potential. A comprehensive understanding of natural product biosynthesis not only deepens our knowledge of their chemical complexity but also drives advancements in chemical synthesis and metabolic engineering, paving the way for the generation of novel bioactive compounds. In this study, we report that a marine axenic culture of *B. subtilis* LP synthesizes six turnagainolides (**1–6**), which exhibit both biofilm-inhibitory and cytotoxic activities. These findings expand our understanding of the structure-activity relationships of turnagainolides and offer new insights into their potential biological roles. Moreover, the identification of biosynthetic gene clusters and the proposed biosynthetic pathway provide a valuable framework for elucidating turnagainolide biosynthesis, laying the groundwork for future efforts to optimize their production and explore their applications in drug development.

## INTRODUCTION

Microbial natural products are renowned for their diverse biological activities and intricate structures, making them an enduring and invaluable source for new drug leads. However, directly linking microbial natural products to their biosynthetic gene clusters remains a significant challenge, with the underlying biosynthetic mechanisms often elusive. This gap in knowledge hampers the effective development and utilization of microbial natural products. As of 2024, approximately 36,545 microbial natural products have been added to the database of The Natural Product Atlas (1). Despite this impressive repertoire, the Minimum Information about a Biosynthetic Gene Cluster (MIBiG) database currently documents only around 2,500 biosynthetic gene clusters associated with microorganisms (2). This discrepancy highlights the urgent need to explore and characterize the biosynthetic gene clusters of microbial natural products and elucidate their biosynthetic pathway.

Turnagainolides are a distinctive family of cyclic depsipeptides containing one (*E*)-3-hydroxy-5-phenylpent-4-enoic acid (Hppa) and four proteinogenic amino acids (Fig. S1). As the first member of this family, compound EGM-556 was discovered from the marine fungus *Microascus* sp. EGM-556 following the introduction of the histone deacetylase inhibitor suberoylanilide hydroxamic acid to activate a silent biosynthetic gene cluster. However, its stereochemistry was only partially resolved, and no bioactivity was reported (3). Subsequently, turnagainolides A and B were identified from *Bacillus* sp., with turnagainolide B functioning as an activator of the SHIP1 enzyme *in vitro*, showcasing potential as a drug candidate for treating inflammatory disorders and hematologic cancers (4). Further studies uncovered additional congeners of turnagainolides from actinomycete strains. For instance, Igarashi et al.reported arthroamide and turnagainolide A from *Arthrobacter* sp., both demonstrating quorum-sensing inhibition in *Staphylococcus aureus* with IC_50_ values of 0.3 µM and 0.8 µM, respectively (5). The structural variation between turnagainolide A and arthroamide arises from the substitution of l-Ile-4 with l-Val-4 (Fig. S1). Another congener, strepeptolide, was discovered from an endophytic *Streptomyces* strain, although its stereochemistry remains unresolved to date (6). The most recent member, turnagainolide C, was identified from *Streptomyces* sp. S2236 and demonstrated anti-microbial activity against *Candida albicans*, *S. aureus*, and *Escherichia coli* (7). Collectively, these studies reveal that turnagainolides and their congeners not only exhibit diverse chemical structures but also possess a range of bioactivities.

Despite their identification across multiple microbial lineages, the biosynthetic gene cluster of turnagainolides has remained elusive, leaving a critical knowledge gap in understanding their biosynthesis. In this study, we screened microbial strains in our laboratory and identified six turnagainolides (**1–6**) from *B. subtilis* LP. Through an integrated approach involving phylogenetic analysis, genome sequencing, anti-SMASH-based prediction, gene knockout experiments, and comparative analyses, we successfully identified the biosynthetic gene cluster for turnagainolides and proposed a putative biosynthetic pathway. Our findings not only enrich the chemical diversity of cyclic peptides in *Bacillus* but also lay a solid foundation for future research on the biosynthesis, bioengineering, and potential applications of turnagainolides.

## RESULTS

### Identification of turnagainolides

**Fig 1:**
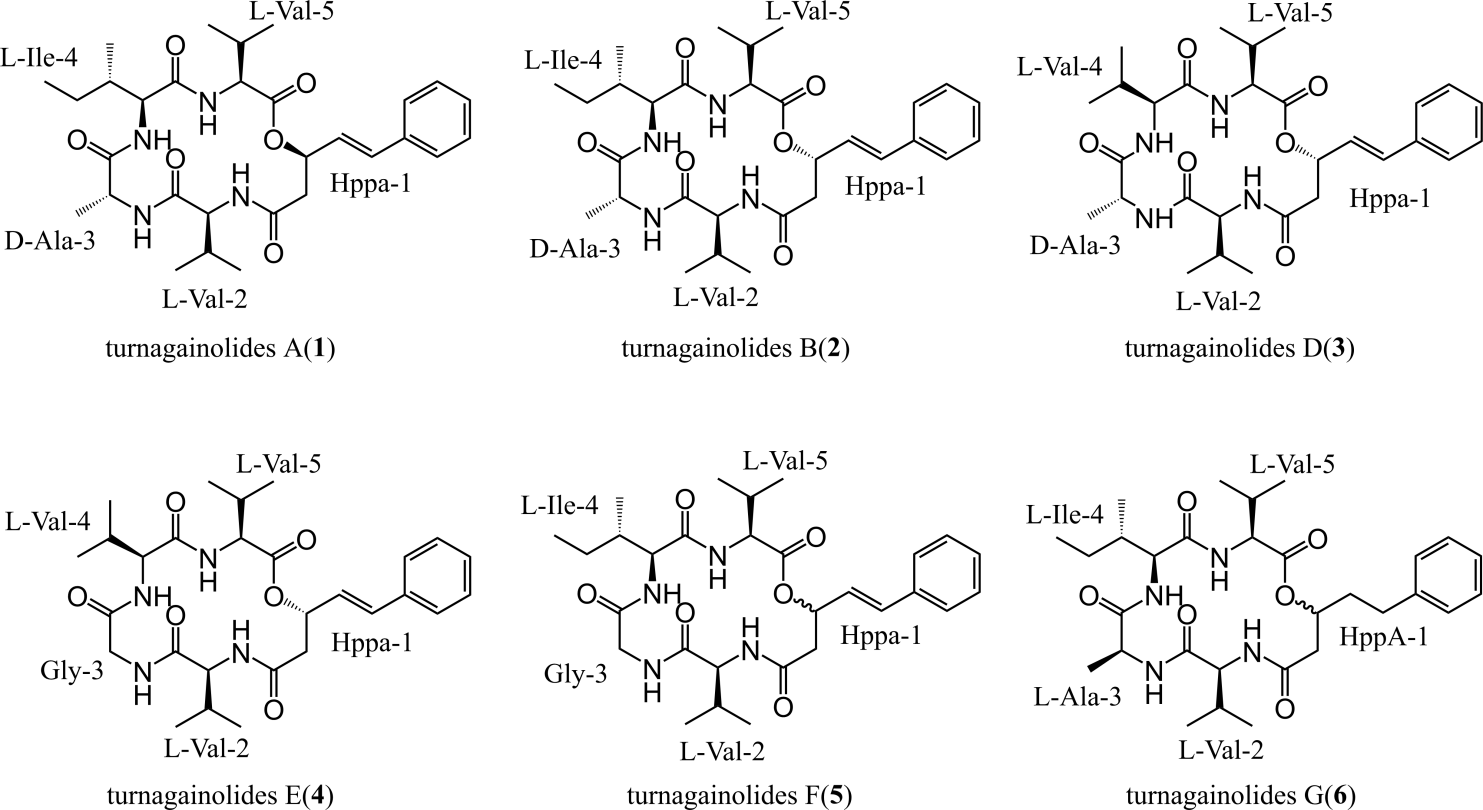
The structures of turnagainolide A and B (**1, 2**) and D–G (**3–6**) are listed with the names of residues.

The strain LP was isolated from a sponge sample collected from the Xisha District (China) and designed as *B. subtilis* through 16S rRNA gene analysis (Fig. S2). LC-MS analysis of strain fermentation extract revealed a series of UV peaks detected at 210 nm between 5 and 7 min (Fig. S3). These peaks shared a characteristic UV absorption at 190 nm and 251 nm, as well as consistent ion patterns as known turnagainolides (Fig. S4 and S5) (4). To elucidate the structures of these compounds, semi-preparative high-performance liquid chromatography (HPLC) coupled with a silica gel column was employed for compound isolation and purification. Ultimately, six pure compounds were obtained (Fig. 1).

**Fig 2:**
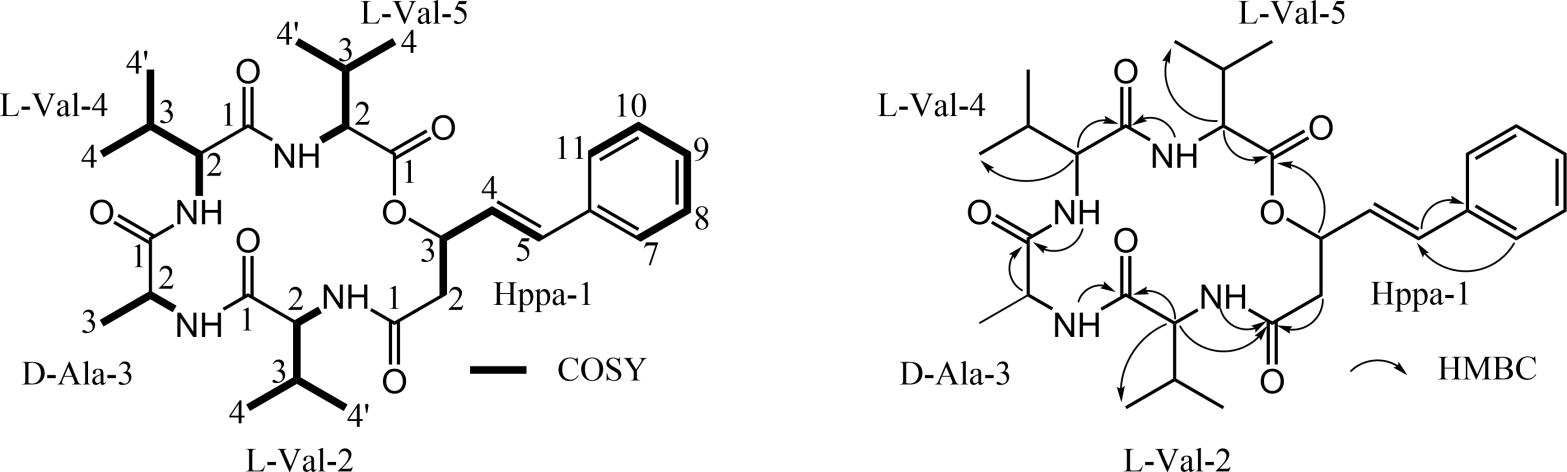
Key 2D NMR correlations of turnagainolide D (**3**).

Turnagainolide D (**3**) was isolated as a white powder and gave a molecular formula of C₂₉H₄₂O₆N₄ based on a [M + H]^+^ ion at *m/z* 543.3188 (calcd for C_29_H_43_O_6_N_4_, 543.3183, error in Δm = 0.9 ppm) in the HRESIMS spectrum (Fig. S5), indicating 11 degrees of unsaturation. The ¹³C, HSQC NMR data analysis revealed the presence of 29 carbons, including five carbonyl carbons, one sp^2^ quaternary carbon, seven sp^2^ methines, eight sp^3^ methines, one sp^3^ methylene, and seven methyls (Fig. S6). In the ^1^H NMR spectrum, a monosubstituted benzene was observed at *δ*_H_ 7.28, 7.35, and 7.44 (Table 1). In addition, four doublet protons (*δ*_H_ 7.58, 7.76, 8.13, and 8.56) were detected and assigned as the amide NH protons since they had no HSQC correlations while showing COSY correlations to the amino acid *α*-protons. The detailed analysis of the COSY, HSQC, and HMBC data led to the routine identification of Val-2, Ala-3, Val-4, and Val-5, and they were linked sequentially (Fig. 2). In Hppa residue, the H-7 and H-11 had HMBC correlations to C-5, and the H-5 had HMBC correlations to C-6. The substructure from C-2 to C-5 in Hppa bearing one ester was confirmed by sequential COSY correlations. In addition, HMBC correlations were observed from H-3 and H2 to the carbonyl carbon at *δ*_C_ 168.83 (C-1 in Val-5) and *δ*_C_ 168.77 (C-1 in Hppa), respectively. These correlation data established the presence of a 3-hydroxy-5-phenyl-4-pentenoic acid (Hppa) unit. Furthermore, the HMBC correlations of NH (Val-2) with C-1 (Hppa-1) and H-3 (Hppa-1) with C-1 (Val-5) indicated that the Hppa residue is located between Val-2 and Val-5, forming a cyclic depsipeptide via ester and amide bonds. LC-MS/MS analysis confirmed this structure through a fragmentation pattern consistent with the proposed structure (Fig. S7).

**TABLE 1:** The ^1^H and ^13^C NMR data of turnagainolide D (**3**), E (**4**), F (**5**), and G (**6**) in DMSO-*d6*

Residue	Position	3*δ*_C_	3*δ*_H_	4*δ*_C_	4*δ*_H_	5*δ*_H_	6*δ*_C_	6*δ*_H_
Hppa-1	1	77		93			169.92	
	2	40.11	2.90, dd (14.2, 11.4) 2.41, dd (14.2, 2.4)	40.06	2.91, dd (14.8, 11.4) 2.45, dd (14.8, 2.4)	2.90, dd (14.8, 11.4) 2.45, dd (14.8, 2.4)	40.40	2.38, m 2.66, m
	3	73.08	5.49, m	72.73	5.55, m	5.52, m	73.13	4.97, m
	4	126.71	6.28, dd (16.0, 7.2)	126.71	6.27, dd (16.0, 7.2)	6.29, dd (16.0, 7.2)	36.11	1.80, m 1.96, m
	5	132.57	6.68, d (16.0)	132.55	6.68, d (16.0)	6.68, d (16.0)	31.25	2.61, m
	6	135.76		135.76			141.56	
	7,11	126.57	7.44, d (7.6)	126.54	7.44, d (7.6)	7.44, d (7.6)	128.67	7.28, m
	8,10	128.76	7.35, t (7.6)	128.75	7.35, t (7.6)	7.35, t (7.6)	128.87	7.18, m
	9	128.21	7.28, t (7.6)	128.18	7.28, t (7.6)	7.28, t (7.6)	126.41	7.17, m
Val-2	1	172.43		170.44			172.85	
	2	57.56	4.13, dd (8.6,6.6)	58.38	4.24, t (9.4)	4.12, m	57.47	4.16, dd (8.8, 6.6)
	3	29.90	1.97, m	29.05	2.20, m	1.99, m	30.70	1.92, m
	4	18.25	0.89, m	18.92	0.88, m	0.88, m	12.41	0.84, m
	4’	18.93	0.89, m	19.32	0.88, m	0.88, m	16.00	0.84, m
	NH		7.76, d (8.6)		7.72, d (8.4)	7.81, d (8.4)		7.47, d (8.8)
Gly-3 (Ala-3)	1	173.25		169.73			173.62	
	2	48.93	4.33, m	43.52	3.43, m 3.97, m	3.36, m 3.81, dd (14.0, 5.2)	49.31	4.29, m
	3	16.39	1.19, d (6.8)				16.72	1.17, d (6.8)
	NH		8.56, d (5.8)		8.87, d (5.8)	8.78, d (5.8)		8.55, d (5.8)
Val-4 (Ile-4)	1	170.52		172.58			171.03	
	2	57.35	4.23, m	58.03	4.10, dd (8.4, 6.6)	4.21, t (9.7)	57.97	4.22, dd (9.4, 4.3)
	3	28.59	2.37, m	28.94	2.31, m	2.22, m	35.96	2.03, m
	4	19.15	0.83, m	19.44	0.84, m	0.82, m	19.81	0.84, m
	4’	16.65	0.83, m	19.40	0.84, m	1.22, m	24.08	1.27, m
	5					0.82, m	20.44	0.84, m
	NH		8.13, d (9.6)		7.94, m	8.39, d (8.5)		8.11, d (9.6)
Val-5	1	168.83		168.93			169.53	
	2	58.36	4.26, dd (9.6, 5.2)	58.24	4.17, dd (9.6, 5.2)	4.11, m	57.88	4.31, m
	3	28.45	2.25, m	29.73	2.00, m	1.56, m,	28.82	2.22, m
	4	19.67	0.89, m	18.15	0.88, m	0.88, m	19.19	0.92, d (6.6)
	4’	19.45	0.89, m	17.13	0.88, m	0.88, m	18.46	0.92, d (6.6)
	NH		7.58, d (9.6)		8.35, d (9.6)	7.51, d (9.6)		7.58, d (9.6)

Turnagainolide E (**4**) was obtained as a white powder that gave a [M + H]^+^ ion at *m/z* 529.3026 (calcd for C_28_H_41_O_6_N_4_, 529.3026, error in Δm = 0.0 ppm) in the HRESIMS spectrum (Fig. S5), consistent with a molecular formula of C_28_H_40_O_6_N_4_. A comprehensive analysis of NMR data revealed a scaffold similar to that of **3**, except for the substitution of Ala-3 with Gly-3 (Table 1; Fig. 1; Fig. S8), which was further supported by comparing LC-MS/MS ion fragments (Fig. S7).

Turnagainolide F (**5**) was isolated as a white powder and yielded a [M + H]^+^ ion at *m/z* 543.3173 (calcd for C_29_H_42_O_6_N_4_, 543.3183, error in Δm = 1.8 ppm) in the HRESIMS spectrum (Fig. S5), consistent with a molecular formula of C_29_H_42_O_6_N_4_. However, the amount was insufficient to collect a full set of NMR data. Therefore, we only collected ^1^H and COSY NMR (Table 1; Fig. S9) data and introduced LC-MS/MS analysis (Fig. S7) to compare the fragment pattern with compound **4**. The results confirmed that compound **5** was similar to **4**, except for the substitution of Val-4 with Ile-4 (Fig. 1).

Turnagainolide G (**6**) was obtained as a white powder and yielded a [M + H]^+^ ion at *m/z* 559.3491 (calcd for C_30_H_46_O_6_N_4_, 559.3496, error in Δm = 0.9 ppm) in the HRESIMS spectrum (Fig. S5), consistent with a molecular formula of C_30_H_46_N_4_O_6_. The NMR data revealed a scaffold similar to **3** except for the reduction of the double bond between C-4 and C-5 in Hppa-1 residue and the substitution of Val-4 with Ile-4 (Table 1; Fig. 1; Fig. S10).

Turnagainolide A (**1**) and B (**2**) were confirmed to be identical to previously reported data (4). They exhibited [M + H]^+^ ions at m/z 557.3324 and 557.3328 (calcd for C₃₀H₄₅O₆N₄, 557.3339, error Δm = 2.7 and 2.0 ppm, respectively) (Fig. S5). Structural confirmation was achieved using NMR and LC-MS/MS analysis data (Fig. 1; Table S1; Fig. S11 and S12).

**Fig 3:**
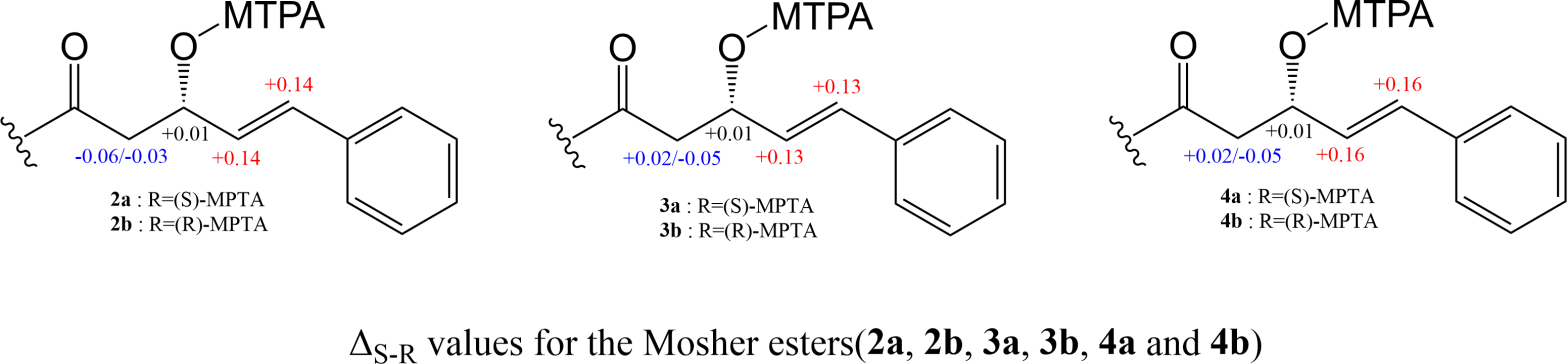
Mosher ester analysis of the Hppa residue in **2**, **3**, and **4**.

To determine the stereochemistry of compounds **1–6**, we first used Marfey’s analysis to establish the configurations of the amino acids. Our results showed that the amino acids in compounds **1–6** were predominantly l-configured, with the exception of Ala-3 in **1–3**, which exhibited a d-configuration (Fig. S13). Subsequently, we utilized NaOMe to catalyze methanolysis of the ester linkage in turnagainolides, exposing the hydroxyl group. Mosher ester analysis was then employed to establish the configuration at C-3 (Hppa-1) (Fig. 3, and S14-S16). The configuration of C-3 in the Hppa residue was determined as *S* in compounds **2–4**. Since compound **2** has the same planar structure as compound **1** and the configuration of four amino acids is the same, the C-3 of the Hppa in compound **1** is considered as *R*, which matches well with the previous report (4). However, it could not be established for **5** and **6** due to the limited sample availability. The complete structures of **1–6** are depicted in Fig. 1. After the structure elucidation and screening of similar compounds in the SCI-Finder database, we found six known compounds, namely arthroamide, strepeptolide, turnagainolides A-C, and EGM-556, exhibited high structural similarity to the isolated turnagainolides (Fig. S1). The structural variation mainly comes from the substitution of the 4th amino acid and stereochemistry of Hppa and Val-2. Interestingly, all six known compounds were identified from distinct microbial species, including *Microascus*, *Arthrobacteria*, *Streptomyces*, and *Bacillus*.

### Identification of turnagainolide BGC

To explore the biosynthetic gene cluster (BGC) of turnagainolides, we sequenced the genome of *B. subtilis* LP and used anti-SMASH for secondary metabolite BGC prediction. This analysis revealed a total of 12 biosynthetic gene clusters (Fig. S17). Given the structural composition of turnagainolides, which includes four proteinogenic amino acids and one Hppa residue, turnagainolides were hypothesized to be synthesized via a multi-modular nonribosomal peptide synthetase (NRPS) pathway.

**Fig 4:**
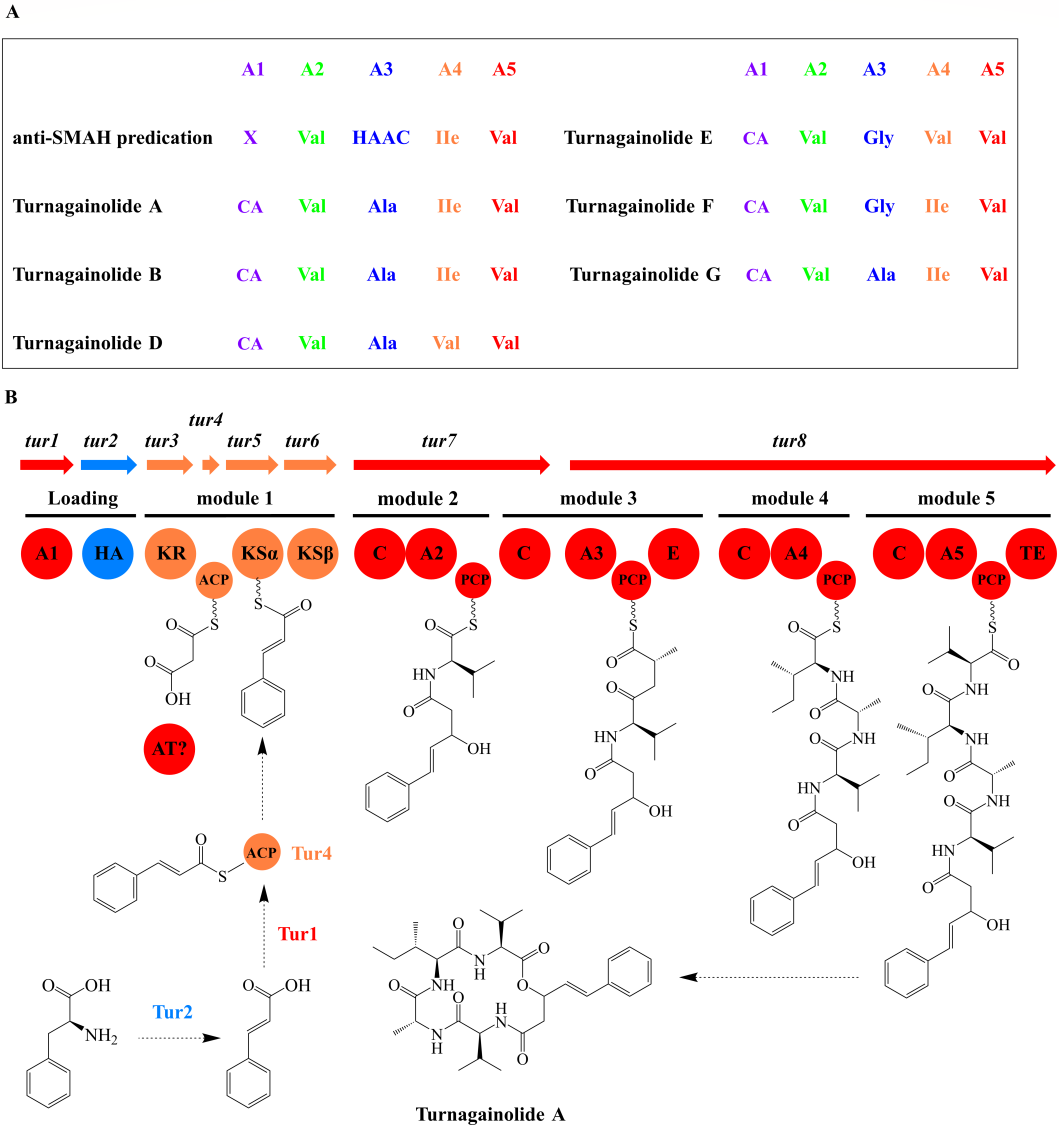
The proposed biosynthetic pathway of turnagainolide A. (**A**) The amino acids activated by the adenylation domains. The “d-” indicates the d-configuration, while the other amino acids exhibit the l-configuration. CA: cinnamic acid, HAAC: hydrophobic-aliphatic amino acid. (**B**) The putative biosynthesis pathway of turnagainolide A. Abbreviations: A, adenylation domain; ACP, acyl carrier protein in PKS; C, condensation domain; E, epimerase domain; HA, histidine ammonia-lyase domain; KSα, keto-synthase domain; KSβ, keto-synthase domain; KR, keto-reductase domain; PCP, peptidyl-carrier protein; TE, thioesterase domain.

During bioinformatic analysis, we identified a 22,529 bp gene cluster comprising eight open reading frames (ORFs) (Table 2). The *tur1* and *tur3-8* encode five NRPS modules and one type II polyketide synthase (PKS) module. The substrate specificity of the adenylation domains within the cluster is consistent with the amino acid residues found in turnagainolides, strictly following the collinearity rule (Fig. 4A) (8). Based on these findings, the gene cluster was designated as BGC-*tur*. To establish the link between BGC-*tur* and turnagainolides, we employed CRISPR/Cas9-based homologous recombination to knock out the *tur7* gene. Subsequent HPLC analysis of the mutant against the wild-type strain revealed the absence of turnagainolides in the fermentation extracts (Fig. S18), confirming that the *tur* cluster is essential for their biosynthesis. Comparative sequence analysis of proteins encoded by the *tur* cluster with the NCBI non-redundant protein database indicated that homologous sequences are widely distributed within the *Bacillus* genus (Fig. S19).

**TABLE 2:** Gene details of BGC-*tur*

Gene name	Gene function	Identity	Organism/GenBank accession no.
*tur1*	AMP-dependent synthetase and ligase	98.58%	*B. spizizenii*/WP_061186915.1
*tur2*	Histidine ammonia-lyase	99.08%	*B. spizizenii*/KXJ38045.1
*tur3*	3-oxoacyl-ACP reductase FabG	71.78%	*B. gaemokensis*/WP_033678927.1
*tur4*	Acyl carrier protein	98.75%	*B. atrophaeus*/WP_033885114.1
*tur5*	Beta-ketoacyl synthase (ladderane biosynthetic-additional SMCOG1022)	99.75%	*B. subtilis*/WP_033885114.1
*tur6*	Beta-ketoacyl synthase (t2fas biosynthetic-additional SMCOG1093)	100%	*B. subtilis*/WP_033885115.1
*tur7*	Non-ribosomal peptide synthetase	99.93%	*B. subtilis*/WP_088326421.1
*tur8*	Non-ribosomal peptide synthetase	100%	*B. subtilis*/WP_259266246.1

According to the collinear relationship between the BGC-*tur* assembly line and the identified structures, we proposed one putative biosynthetic pathway for turnagainolide A. The biosynthesis is considered to begin with the deamination of l-phenylalanine (l-Phe), which is catalyzed by the histidine ammonia-lyase (*tur2*). The resulting cinnamic acid is activated by AMP-dependent synthetase and ligase (*tur1*) and loaded onto the KSα domain (*tur5*) mediated by the ACP domain (*tur4*), while one malonyl-CoA is subsequently transferred to the ACP domain again by one unknown acyltransferase domain. The KSα domain mediates the condensation of cinnamic acid with malonyl-CoA. The keto-reductase (KR) domain exhibits 71.78% identity to FabG (9), which likely determines the configuration of the C-3 in Hppa. The initiation and two-carbon unit extension exhibit a great similarity to the biosynthetic part of siderochelin (10). The NRPS Modules in Tur7 and Tur8 were predicted to add Val-2, X-3, Ile-4, and Val-5 into the growing chain in order. Analysis of the A3 domain suggests specificity for one hydrophobic-aliphatic residue, consistent with the structural diversity observed at the third residue position in turnagainolides (Fig. 1). Notably, module 3 contains an epimerase (E) domain, suggesting the incorporation of a d-amino acid, which aligns with the presence of d-Ala at the third residue position in compounds **1–3**. This provides strong evidence for the active role of the E domain in the biosynthesis pathway. At the end of the biosynthetic assembly line, the thioesterase (TE) domain in Tur8, containing the canonical “GXSXG” motif (11) (Fig. S20), catalyzes the release and cyclization of the linear peptide chain. This forms a macrolactone ring via esterification between the hydroxyl group of Hppa and the carbonyl group of l-Val-5.

### Determination of biological activity

Turnagainolides have been reported to inhibit quorum-sensing signals in *S. aureus*. Since biofilm formation is regulated by quorum sensing in bacteria, we evaluated the anti-biofilm activity of the identified turnagainolides. Compound **1** displayed weak inhibitory activity at a concentration of 400 µg/mL, while compounds **2** and **3** exhibited slightly stronger inhibition at concentrations above 100 µg/ml (Fig. S21). In addition, cytotoxicity assays revealed that compound **4** had significant cytotoxic effects on mouse tumor cell lines BV2 and MB49, with IC_50_ values of 57.0 µM and 88.6 µM, respectively.

## DISCUSSION

Microbial natural products have long been recognized as a rich resource for drug discovery. However, their low production yields often constrain laboratory research and limit their applications across various fields. Since the beginning of the 21st century, advances in sequencing technology and the development of genetic engineering have facilitated the elucidation of an increasing number of biosynthetic pathways for microbial natural products (2). These breakthroughs have provided a solid foundation for enhancing compound production through synthetic biology and metabolic engineering approaches (12, 13). Despite these advancements, only a small fraction of biosynthetic gene clusters (BGCs) has been uncovered. In this study, we focused on identifying the BGC responsible for turnagainolides and proposed their biosynthetic pathway, revealing the chemical diversity caused by enzyme promiscuity and the key role of some enzymes. The discovery of novel turnagainolides and new biological activities not only underscores the significance of investigating turnagainolides but also enriches the repository of cyclic peptides in *Bacillus*.

Turnagainolides represent a class of cyclic depsipeptides characterized by one (*E*)-3-hydroxy-5-phenylpent-4-enoic acid (Hppa) residue and four proteinogenic amino acids in their structures. To date, six congeners have been reported from multiple microbial lineages (3–7). Their structural diversity primarily arises from variations in the 4th amino acid residue and the stereochemistry of Hppa and Val-5 (Fig. S1). In this study, we identified six turnagainolides (**1–6**) from *B. subtilis* LP (Fig. 1), four of which (compounds **3–6**) are novel. The new structures exhibited one more variation on the 3rd amino acid residue, which is occupied by l-Ala-3 or Gly-3 in compounds **4–6**. All those structural features highlight a combination of enzymatic promiscuity and conservativeness in the biosynthesis of turnagainolides.

The identification of turnagainolides from various microorganisms has been reported for some time. While horizontal gene transfer is well documented for microbial natural products, particularly among closely related strains (14–16), the transfer of biosynthetic gene clusters (BGCs) between bacteria and fungi is considered rare. Notably, Li et al. discovered turnagainolides A and B in *Bacillus* species, raising questions about the initial discovery of EGM-556 from *Microascus* species and suggesting a possible association with commensal or symbiotic bacteria (4). Earlier reports largely focused on identifying new compounds without conducting phylogenetic analyses of the producing strains or verifying their axenic cultures (3–7). In this study, we performed strain isolation and purification, phylogenetic analysis, genome sequencing, and gene knockout experiments to directly link the production of turnagainolides to the *tur*-BGC. This provides a valuable framework for exploring turnagainolide structural diversity and biosynthetic mechanisms in other microorganisms. Furthermore, our alignment of enzymes involved in turnagainolide biosynthesis against the NCBI non-redundant protein database revealed hits predominantly in the *Bacillus* genus (Fig. S20), encompassing multiple species, but none in other bacteria or fungi. This finding supports the hypothesis that previous reports of turnagainolides from non-*Bacillus* organisms may be attributable to contamination or the presence of symbiotic *Bacillus*. While further evidence is needed to confirm this hypothesis, our results underscore the need to address unresolved questions regarding the true biological source of turnagainolides.

The identification of the *tur-*BGC adds to the growing repertoire of cyclic peptide gene clusters in *Bacillus*. To date, several cyclic peptides, including surfactin (17), bacillomycin (18), fengycin (19), and iturins (20), have been reported from this genus. These compounds share a macrocyclic peptide ring structure typically composed of seven amino acids and a long-chain fatty acid. By contrast, turnagainolides feature a smaller cyclic peptide ring and lack a long-chain fatty acid. Interestingly, while surfactin, bacillomycin, fengycin, and iturins exhibit potent antibacterial and antifungal activities, turnagainolides only demonstrate moderate inhibition of biofilm formation. This structural difference led us to hypothesize that the long-chain fatty acid plays a crucial role in the antimicrobial activities of other cyclic peptides, but it seems that loss of the long-chain fatty acid attributes different bioactivity to turnagainolides. Despite these differences, all these cyclic peptides share a common biosynthetic feature: they are synthesized via the NRPS assembly line. In our study, we found that the adenylation (A) domains A1, A2, and A5 exhibit strict substrate specificity, activating cinnamic acid (CA), valine (Val), and valine, respectively (Fig. 4A). However, the A3 and A4 domains display significant substrate promiscuity, activating different aliphatic-hydrophobic amino acids. This enzymatic promiscuity suggests potential opportunities for engineering peptide structures by manipulating NRPS modules, such as swapping adenylation domains.

Macrocyclization is one of the most challenging steps in synthetic chemistry (21). However, microorganisms have evolved various strategies to overcome these challenges, including the use of thioesterases in NRPS, PKS, and hybrid assembly lines (22). During our analysis of the turnagainolide biosynthetic pathway, we found a TE domain at the end of the gene cluster, which is predicted to catalyze chain release and cyclization (23). Enzymatic cyclization is considered to offer several advantages over chemical methods, including higher efficiency, specificity, stereoselectivity, and compatibility with mild reaction conditions. By contrast, chemical cyclization often faces significant challenges, such as conformational constraints, side reactions, kinetic and thermodynamic barriers, stereochemical issues, and solubility limitations. For instance, Li et al. successfully synthesized turnagainolides A and B chemically but achieved cyclization yields of only 15% and 33%, respectively(4). Thus, the TE domain discovered in the turnagainolide pathway may provide a promising alternative for addressing the challenges of short peptide cyclization. Interestingly, our analysis also revealed that the *tur*-BGC incorporates a type II PKS module, which adds a two-carbon unit to extend the linear peptide chain prior to cyclization via a lactone bond. While the evolutionary advantage of this extra step remains unclear, we consider it may help reduce spatial constraints and facilitate macrocyclization.

Turnagainolides and their congeners exhibit diverse bioactivities, with structural differences correlating with specific functional properties. For example, turnagainolide B differs from turnagainolide A in the stereochemistry of the Hppa residue, and only turnagainolide B activates SHIP1, suggesting that the *S*-Hppa configuration is critical for bioactivity. Similarly, turnagainolide C, distinguished by a d-Val-5 residue, is the only congener that exhibits antibacterial activity, highlighting the importance of amino acid stereochemistry. In our biofilm inhibition assays, compound **1** showed no activity at 200 µg/mL, while compounds **2** and **3** exhibited weak inhibition at concentrations above 100 µg/mL, further supporting the role of *S*-Hppa in shaping turnagainolide bioactivity. In addition, the anticancer activity detected for compound **4** in this study suggests that turnagainolides may possess a broader range of bioactivities than previously identified, warranting further exploration.

## MATERIALS AND METHODS

### Strain isolation and phylogenetic analysis

A single sponge sample, identified as *Plakortis halichondrioides* XSSP-8, was collected from a mesophotic coral reef at a depth of 18 m in the Xisha District of China (16°N, 111°E). The sample (2 g) was washed with distilled water, finely minced with a scalpel, and ground in liquid nitrogen. The resulting homogenate was mixed with 1 mL of 50% glycerol and serially diluted 10-fold in the same solution. A 50 µL aliquot of the diluted suspension was spread onto agar plates containing 30% R2A medium supplemented with a combination of nalidixic acid (20 mg/mL) and nystatin (25 mg/mL) or polymyxin (10 mg/mL) and nystatin (25 mg/mL), respectively. Sterilized glass beads (8–10 beads, 5 mm in diameter) were used to evenly distribute the inoculum. The plates were incubated at 26℃ for 7 days, allowing for the isolation of target colonies. Single colonies were further purified by streaking onto fresh agar plates. Axenic cultures were confirmed by colony PCR targeting the 16S rRNA gene, using universal primers 27F (5′-AGAGTTTGATCCTGGCTCAG-3′) and 1492R (5′-GGTTACCTTGTTACGACTT-3′). PCR conditions included an initial denaturation at 95℃ for 5 min, followed by 30 cycles of 95℃ for 30 s, 55℃ for 30 s, and 72℃ for 2 min, with a final extension at 72℃ for 10 min. The amplified 16S rRNA gene products were sequenced through the ABI 3730 platform by GENEWIZ (Suzhou, China). Taxonomic identification was performed via BLAST analysis against the GenBank database.

### Fermentation

Seed cultures of *B. subtilis* LP were initiated in 1 L Erlenmeyer flasks containing 400 mL of 100% R2A liquid medium (Hopebio). These cultures were grown at 28℃ with shaking at 160 rpm for 2 days. The seed cultures were then used to inoculate 125 Erlenmeyer flasks (1 L capacity) at 1% (vol/vol), each containing 400 mL of 100% R2A medium and 2% (wt/vol) macroporous adsorbent resin HP-20 (Solarbio). The large-scale fermentation was conducted at 28℃ with shaking at 160 rpm for 7 days.

### Compound isolation and purification

After fermentation, the HP-20 resin beads were harvested by filtration, rinsed with distilled water, and air-dried for 24 hours. The dried resin beads were washed with 100% methanol until no compound residue remained. The methanol extracts were concentrated using a rotary evaporator under reduced pressure, yielding 13 g of crude extract. The crude extract was subjected to open-column chromatography on silica gel (300–400 mesh, column dimensions 50 × 4 cm). Sequential elution was performed with a series of solvent mixtures, including petroleum ether (100%), petroleum ether/ethyl acetate (1:1), ethyl acetate (100%), and ethyl acetate/methanol mixtures in ratios of 9:1, 8:2, 7:3, 1:1, and 3:7, followed by 100% methanol. Each solvent was passed through the column three times, resulting in 17 fractions, which were analyzed by HPLC. For HPLC, samples were injected into a Luna C18 column (100 × 3 mm, 2.6 *µ*m, Phenomenex) with a gradient elution program transitioning from 10% aqueous MeCN to 100% MeCN over 30 min at 25℃ at a flowing speed of 1 mL/min. The turnagainolides were detected in fractions eluted with ethyl acetate/methanol (9:1), which were pooled, evaporated, and further purified by semi-preparative HPLC using a Luna C18 column (250 × 10 mm, 5 *µ*m, Phenomenex) at a flow rate of 2 mL/min. An isocratic elution of 40% aqueous MeCN for 120 min at 25℃ was used, yielding six monomeric compounds: compound **1** (6.2 mg, tR = 95.24 min), compound **2** (17.1 mg, tR = 103.48 min), compound **3** (2.2 mg, tR = 53.92 min), compound **4** (5.8 mg, tR = 68.69 min), compound **5** (0.5 mg, tR = 80.97 min), and compound **6** (0.8 mg, tR = 113.30 min). All solvents were HPLC grade (Fisher Scientific).

### LC-MS analysis

LC-MS analysis was performed using a Waters Xevo G2-XS Quadrupole Time-of-Flight instrument. Samples were separated on a BEH C18 column (100 × 2.1 mm, 1.7 µm, ACQUITY UPLC) using a gradient elution of 10% aqueous MeCN to 100% MeCN over 18 min at a flow rate of 0.3 mL/min, with 0.1% formic acid as a modifier. Electrospray ionization (positive mode) was employed, with a scan range of *m/z* 100–1500. Targeted MS/MS spectra were averaged over three scans, with collision energies of 13 eV (*m/z* 250), 20 eV (*m/z* 350), and 27 eV (*m/z* 450). Instrument settings included an ion source temperature of 200°C, nebulizing gas flow at 3 L/min, heat block temperature at 350°C, and drying gas flow at 15 L/min.

### NMR analysis

NMR spectra were recorded on a Bruker Avance IIIHD 600 MHz spectrometer (600 MHz for ¹H and 150 MHz for ¹³C). Chemical shifts were referenced to residual solvents (dimethyl sulfoxide [DMSO]: ¹H, *δ* 2.50; ¹³C, *δ* 39.52; CDCl_3_: ¹H, *δ* 7.26) and reported relative to tetramethylsilane (TMS).

### Marfey’s derivatization

A total of 0.2 mg of the compound was hydrolyzed with 0.5 mL of 6M HCl at 110°C for 19 hours. After cooling to room temperature, the solvents were removed using a rotary evaporator, and the residue was resuspended in 100 µL of deionized water. Subsequently, 100 µL of 1% l-FDLA (N-(*α*)-(5-fluoro-2,4-dinitrophenyl)-l-leucinamide, ABCR GmbH & Co., Germany) in acetone was added, followed by 20 µL of 1M NaHCO_3_. The reaction mixture was incubated at 37°C for 1 hour, after which the reaction was terminated by adding 20 µL of 1M HCl. Standard amino acid samples were processed using the same protocol. The derivatized samples were analyzed using liquid chromatography-mass spectrometry (LC-MS) with a Q Exactive Mass Spectrometer (Thermo Scientific) equipped with a C18 column (3 µm, 100 × 2.1 mm, Hypersil GOLD). The gradient program ranged from 20% to 80% aqueous acetonitrile over 40 minutes at a flow rate of 0.3 mL/min. For the analysis of Ile, a chiral column (Lux 3 µm i-cellulose-5, 150 × 3.0 mm) was used to separate l-Ile and l-allo-Ile, employing a gradient from 20% to 60% acetonitrile over 90 minutes at a flow rate of 0.4 mL/min.

### Esterification of compounds

To dissolve 1.5 mg of the compound, 2 mL of 5% NaOMe/MeOH was added, and the mixture was stirred at room temperature for 20 minutes. The aqueous phase was neutralized with 2 mL of 1M HCl and extracted three times with equal volumes of ethyl acetate. The organic solvent was removed by rotary evaporation, and the residue was resuspended in 100% methanol. The product was purified using semi-preparative reverse-phase HPLC with isocratic elution at 30% aqueous acetonitrile.

### Mosher derivatization

A total of 0.5 mg of the compound was suspended in 100 µL of pyridine, followed by the addition of 2 µL of (*R*)-MTPA-Cl. The reaction mixture was incubated at room temperature for 10 hours and subsequently purified using semi-preparative reverse-phase HPLC with a C18 column (150 × 4.6 mm, 4 µm, Agilent). The elution was performed using 33% aqueous acetonitrile. An identical procedure was followed for (*S*)-MTPA-Cl. The configurations of the derivatives were analyzed by NMR spectroscopy (Bruker Avance IIIHD 600 MHz) in CDCl_3_.

### Biofilm inhibition experiment

*S. aureus* was cultured overnight in 3 mL of TSB medium. The next morning, the culture was diluted to an OD_570_ of 0.5 with fresh TSB medium and then further diluted 1,000-fold to achieve a final concentration of 1 × 10^6^ CFU/mL. Aliquots of 100 µL of the diluted culture were transferred to individual wells of a 96-well plate. Test compounds were added to the first well of each row at a concentration of 800 µg/mL, followed by twofold serial dilutions across the row. The plate was incubated overnight at 37°C. The medium was subsequently discarded, and the wells were washed three times with PBS (KH_2_PO_4_ 0.27 g, Na_2_HPO_4_ 1.42 g, NaCl 8 g, KCl 0.2 g in 1 L deionized water, pH 7.4). Next, 200 µL of methanol was added to each well to fix the biofilm, and the plate was incubated for 15 minutes. The methanol was then removed, and the wells were stained with 0.1% crystal violet for 5 minutes. Excess dye was washed off, and the plate was air-dried. To dissolve the bound stain, 200 µL of glacial acetic acid was added to each well, and the plate was shaken for 20 minutes. The absorbance at OD_570_ was measured using a microplate reader (Thermo Fisher Scientific, Varioskan LUX).

### Cytotoxicity test

The cytotoxicity of curcumin was evaluated using an MTT-based colorimetric assay. Cells were seeded in 96-well plates at a density of 5 × 10^3^ cells per well. After 24 hours, 500 µL of 5 mg/mL MTT solution was added to each well. Following 3 hours of incubation at 37°C in 5% CO_2_, the supernatant was removed, and the formazan crystals were dissolved in 100 µL of DMSO. Absorbance at 570 nm was measured using a microplate reader (Wallac 1420, PerkinElmer).

### Genome sequencing and anti-SMASH prediction

Genomic DNA from *B. subtilis* LP was extracted from an 18 hour culture using the Magen HiPure Bacterial DNA Kit. Genome sequencing and assembly were performed by GENEWIZ (Suzhou, China) using the sequencing platform of Nanopore and Illumina platform. The genomic sequence (GenBank accession no. CP152362) was analyzed using the default parameters of the web-based anti-SMASH software.

### Gene knockout

The gene knock-out method followed the protocol of Altenbuchner Josef (24). Primers used in this study are listed in Additional File: Table S2. The sgRNA sequence for the *tur7* target site was designed using the CHOPCHOP tool (http://chopchop.cbu.uib.no/), and the target sequence was GGTACAGCATTAACACCATG. sgRNA fragments were synthesized using primers N20-F and N20-R by denaturation at 95°C for 2 minutes, followed by slow cooling to room temperature. Using *B. subtilis* LP genomic DNA as a template, UP and DOWN fragments flanking the target site were amplified with primers tur7-UP-F, tur7-UP-R, tur7-DOWN-F, and tur7-DOWN-R. After gel purification, UP and DOWN fragments were fused by overlap PCR using primers tur7-UP-F and tur7-DOWN-R to produce a UD fragment. The UD fragment was ligated into the pJOE8999 plasmid via homologous recombination. Transformants were verified using colony PCR with primers PJF and PJR, which yielded a band of 3314 bp. The knockout plasmid was transformed into *B. subtilis* LP, and transformants were confirmed by colony PCR with primers tur7-1 and tur7-2. The wild-type band was 5,926 bp, while the successfully knocked-out band was 3,099 bp (Fig. S22). The strains of LP and mutant were grown at 28℃ with shaking at 160 rpm for 10 days and extracted with ethyl acetate. The extracts were injected into an InfinityLab Poroshell 120 EC-C18 column (150 × 4.6 mm, 4 µm) with a gradient elution program transitioning from 35% aqueous MeCN to 50% MeCN over 45 min at 25℃ at a flowing speed of 0.7 mL/min. The detective wavelength of HPLC analysis is 250 nm.

## Data Availability

All supplementary data, including Fig. S1 to S22 and Table S1 and S2, are publicly available at https://doi.org/10.6084/m9.figshare.28642532.
